# An Unusual Case of Urinary Tract Infection in a Pregnant Woman With *Photobacterium damsela*

**DOI:** 10.1155/IDOG/2006/80682

**Published:** 2006-09-28

**Authors:** Jesus R. Alvarez, Sangeeta Lamba, Keisha Y. Dyer, Joseph J. Apuzzio

**Affiliations:** Obstetrics, Gynecology, and Women's Health Department, University of Medicine and Dentistry of New Jersey, Newark, NJ 07103, USA

## Abstract

We describe a case of a urinary tract infection with an unusual pathogen, Photobacterium damsela, in a pregnant female. This pathogen has been described as having a virulent life threatening nature, so a detailed history and prompt treatment is needed.

## CASE REPORT

We report what is to our knowledge the first case of a urinary tract infection caused by *Photobacterium damsela*, a virulent pathogen, in a 22-year-old gravid female at 23-week gestation.

The patient initially presented for routine prenatal care. At this time she reported frequency and dysuria with no other complaints. She denied any significant past medical history. The only finding on physical examination was suprapubic tenderness and leukocytosis was noted on the urine dipstick. A urine culture was obtained and the patient was treated empirically with Cephalexin 500 mg qid for one week. The urine culture subsequently demonstrated growth > 100000 colonies of *Photobacterium damsela*, which was sensitive to cephalosporins, trimethoprim-sulfamethoxazole, and aminoglycosides. Given the rarity of infection with this pathogen, a more detailed history was obtained where the patient stated have had sexual intercoursein the Caribbean Sea off the coast of Puerto Rico, one week prior to her office visit.

One week after having her first office visit and the urine culture with the above findings, the patient presented to labor and delivery complaining of fever, chills, and foul smelling urine at 24-week gestation. On the day prior to admission, she noted that her urine was malodorous. She complained of suprapubic tenderness and mild right flank pain. On examination she was noted to have an oral temperature of 101.6 and suprapubic tenderness, no costovertebral angle tenderness was noted. No contractions were palpated and on vaginal examination a closed uneffaced cervical os was noted. The white blood cell count was 8800 with 79% polymorphonuclear lymphocytes and no bandemia was present. A urinalysis showed trace blood, positive nitrites, and some bacteria. The fetal heart rate was reassuring and no contractions were noted on the tocometer.

Given the history of the unusual pathogen that grew a week prior to this presentation, the patient was admitted with the diagnosis of pyelonephritis in pregnancy and was started on Cefazolin 2 g IV every 6 hours, and Gentamicin 120 mg IV every 8 hours. The patient remained febrile for 24 hours at which time she had her last temperature spike of 101.6 and fetal tachycardia was noted to 180 beats per minute. She was maintained on the aforementioned antibiotic therapy and defervesced. A complete blood count was repeated on hospital admission day number 3 and her white blood cell count remained within normal limits. On admission day 3 the peak and trough for gentamicin showed therapeutic levels of the antibiotic. No growth was observed on the blood cultures done on admission. The patient was discharged home on hospital day number five. She was advised to take Bactrim for one week followed by suppressive therapy with Macrobid for the remainder of the pregnancy. Urine cultures were repeated monthly for the next 3 months and all remained negative.

## DISCUSSION

Vibrionaceae are classified into six genera, namely, Vibrio, Allomonas, Enhydrobacter, Listonella, Photobacterium, and Salinivibrio. All Vibrio species are common to marine environments, abundant in coastal waters, and have large increases in cell densities in warmer months (particularly in temperate climates), correlating with corresponding increases in disease prevalence. Some of the Vibrio species (V cholerae, V parahaemolyticus, V fluvialis, V furnissii, V hollisae, and V mimicus) are primarily associated with diarrheal disease whereas others cause wound infections (V alginolyticus, V damsela) [1].

*Photobacterium damsela* was first identified in 1981. Initially, classified as a member of the *Vibrio* species which was known to cause death in immunocompromised hosts, it was subsequently designated as *Listonella damsela.* Ultimately this organism was reclassified based on phenotypic data as *Photobacterium*. This is a facultative anaerobic gram-negative bacillus that is pathogenic in both marine life and humans. Its habitat is primarily warm seawater and is most abundant during the summer months. *P* damsela is a known pathogen causing skin ulcers in fish, probably deriving its name from its common victim, the damselfish Chromis punctipinnis. Photobacterium damselae subsp. damselae isolates are known to cause disease in mice and have the capacity to produce large amounts of a cytotoxin (damselysin) with activity against mice erythrocytes in vitro [2, 3]. Damselysin is a phospholipase-D whose activity has been clearly demonstrated in mice and no homologous DNA sequence has been isolated in the other *Vibrio* species [4].

The damage caused by this bacterium has been suggested to be attributable more to the cytotoxin than to the bacterium itself. More recently, Osorio et al, using polymerase chain reaction techniques, concluded that the dly gene is not a prerequisite for pathogenicity, and therefore the role of damselysin as the main virulence factor should be reevaluated [5].

Several cases have been described in the literature of *Photobacterium damsela* as a cause of necrotizing fasciitis in humans [6, 7]. Given the potentially life threatening nature of this infection, early recognition is essential and has been emphasized in the literature. The primary route of infection is minor wounds inflicted by marine life in brackish water with subsequent bacterial inoculation. Contamination of preexisting wounds has also been described. The progression of the disease described in the literature constitutes an initial cellulitis with rapid progression advancing to septic shock and occasions the infected patients may present with relatively inconspicuous skin lesions consistent with an uncomplicated cellulitis and often outpatient antibiotic therapies were prescribed. Patients subsequently return with bulla, subcutaneous hemorrhage, and acute systemic illness with 36 hours of initial evaluation.

Knight et al described a case of bacteremia secondary to Photobacterium damsela in the Caribbean in a child with sickle cell disease [8].

We describe the first case of urinary tract infection secondary to *Photobacterium damsela*. It is also the first case report of a pregnant patient with *P* damsela infection. This isolate was the only pathogen cultured from urine obtained routinely in a symptomatic pregnant, otherwise healthy woman. We can hypothesize that the history of sexual intercourse in the Caribbean coastal waters of Puerto Rico during the summer, one week prior to becoming symptomatic with dysuria and frequency, provided the portal of entry for the pathogen.

Sexual intercourse and pregnancy are among the most common risk factors for urinary tract infections in young women. *E coli* accounts for approximately 80% of all uncomplicated UTIs. Other pathogens include *Klebsiella, Enterobacter, Serratia, Proteus, Pseudomonas, Providencia, Moraganell, Staphylococci, Streptococci, Enterococcus faecalis, Chlamydia, and Candida*. However, urinary tract infection with Photobacterium damsela has never been previously described in the literature. Moreover, other reports have documented this organism's resistance to ampicillin, as was the case with our isolate. Given the high rate of resistance (approximately 30%), ampicillin's use for the treatment of urinary tract infections is not advised. In addition this choice appears to be definitively ineffective in treating this particular pathogen.

The patient was admitted with the diagnosis of pyel-onephritis one week after receiving the empiric therapy with Cephalexin, and was started on Cefazolin IV and Gentamicin IV for which the isolate was sensitive. Blood cultures were negative on admission, where as per the previous cases reported, bacteremia with this pathogen may become lethal producing multiorgan failure. Her early presentation to a physician secondary to symptoms and her pregnant status, along with early empiric therapy with Cephalexin, to which the isolate was sensitive, may have contributed to the relatively benign course in our patient. The effect of urine pH and its bacteriostatic properties and the natural resistance of bladder mucosa to infection may have contributed to decreased bacterial proliferation or decreased production of cytotoxin, hence minimizing the virulence.

Even though this patient had a relatively indolent course, given the pathogenesis of this organism as previously described in the literature; the possibility of life-threatening sequelae should prompt aggressive therapy if *Photobacterium damsela* infection is suspected.
